# A Neuroimaging Study of Personality Traits and Self-Reflection

**DOI:** 10.3390/bs9110112

**Published:** 2019-11-05

**Authors:** Joseph Ciorciari, John Gountas, Patrick Johnston, David Crewther, Matthew Hughes

**Affiliations:** 1 Department of Psychological Sciences, Centre for Mental Health, Swinburne University of Technology, Melbourne 3122, Australia; matthewhughes@swin.edu.au; 2 Department of Psychological Sciences, Adjunct, Swinburne University of Technology and Department of Marketing, Adjunct University of Notre Dame Western Australia, Fremantle 6959, Australia; j.gountas@outlook.com; 3 Faculty of Health, School of Psychology and Counselling, Queensland University of Technology, Brisbane 4000, Australia; patrick.johnston@qut.edu.au; 4 Centre for Human Psychopharmacology, Swinburne University of Technology, Melbourne 3122, Australia; dcrewther@swin.edu.au

**Keywords:** neuroimaging, personality, orientations, self-reflection, thinking styles, fMRI

## Abstract

This study examines the blood-oxygen level dependent (BOLD) activation of the brain associated with the four distinctive thinking styles associated with the four personality orientations of the Gountas Personality Orientations (GPO) survey: Emotion/Feeling-Action, Material/Pragmatic, Intuitive/Imaginative, and Thinking/Logical. The theoretical postulation is that each of the four personality orientations has a dominant (primary) thinking style and a shadow (secondary) thinking style/trait. The participants (N = 40) were initially surveyed to determine their dominant (primary) and secondary thinking styles. Based on participant responses, equal numbers of each dominant thinking style were selected for neuroimaging using a unique fMRI cognitive activation paradigm. The neuroimaging data support the general theoretical hypothesis of the existence of four different BOLD activation patterns, associated with each of the four thinking styles. The fMRI data analysis suggests that each thinking style may have its own cognitive activation system, involving the frontal ventromedial, posterior medial, parietal, motor, and orbitofrontal cortex. The data also suggest that there is a left hemisphere relationship for the *Material/Pragmatic* and *Thinking/Logical* styles and a right activation relationship for *Emotional/Feeling* and *Intuitive/Imaginative* styles. Additionally, the unique self-reflection paradigm demonstrated that perception of self or self-image, may be influenced by personality type; a finding of potentially far-reaching implications.

## 1. Introduction

Understanding the neural basis of human personality is a core imperative of cognitive neuroscience [1,2] and social neuroscience [3,4]. Researchers have employed a variety of techniques including functional magnetic resonance imaging (fMRI) [5], electroencephalography (EEG) [6], in addition to behavioral and psychometric tests [7,8]. One of the many important research questions is to further investigate the neurobiology of behavioral relationships and personality traits/orientations. Several studies have explored these associations between individual personality traits/orientations and decision making [9,10,11]. Personality traits and disorders are found to correlate with poor or maladaptive decision making (e.g., dissociative, compulsive and affective disorders). Maladaptive personality traits and disorders have been associated with deficits in distinct neural systems [12,13,14]. The focus of this study is on the adaptive and ‘normal’ subjects’ personality orientations and putative four thinking styles (GPOs). The aim was to explore the neural basis of the new GPO model using fMRI, and test whether the hypothesized four distinct brain systems’ activations are associated with each of the four personality orientations (GPO). This was achieved with the use of a unique self-reflection fMRI paradigm.

Neuroimaging techniques are well suited to studying how personality traits are coded in the brain because it is non-invasive and affords several relatively simple analytic approaches to reveal spatial localization of discrete functional architectures [15,16]. Personality trait differences activate different brain networks, established through ‘functional connectivity’ of resting-state fMRI acquisitions and inter-regional network connectivities. Using fMRI, Adelstein et al. [17] found that each of the five NEO dimensions predicted distinct brain activations and resting state networks. Haas and colleagues [18] reported that *agreeableness*, a measure of sympathy and social cooperation, predicted blood-oxygen level dependent (BOLD) changes in the temporoparietal cortex during the testing of an emotional attribution task. After controlling for the other Big 5 dimensions, (and educational levels), participants who were highly *agreeable* made faster emotionally valenced decision attributions and exhibited greater BOLD signal intensity in the right temporoparietal junction. The medial frontal cortex (mFC) myelination and activation have also been implicated in other personality studies, respectively [19,20]. In addition, photic driving EEG responses were correlated with the NEOPI-R personality dimensions of *openness*, *agreeableness*, and *consciousness* with multiple EEG bands. The results suggested individual activations in the occipital-parietal and temporal cortices for these three dimensions [21], which have been independently corroborated [22,23]. Several personality related studies have reported that distinct electrical activation characteristics reflect differences in behavioral and decision-making processes associated with specific personality orientations/traits [24,25].

The current neuroimaging study used a Jungian-based personality model, developed by Gountas and colleagues [9,10,26], which identifies four ontogenetically different personality orientations that correspond to different social behavioral preferences and thinking styles. According to Gountas et al. [10], each personality orientation (PO) focuses on different types of information input (e.g., empirical/physical, abstract/pictorial, ideational/conceptual, experiential/ feelings), and consequently, use different cognitive processing systems that affect preferences and decision-making choices. Notably, according to the GPO model, each individual has access to two thinking styles, but one thinking style is the dominant PO (primary) and the second is the secondary PO (e.g., Material dominant PO and Thinking secondary PO). By developing the GPO measure into a word-based self-reflection computer driven experimental task for an EEG, and subsequent fMRI studies, Gountas et al. [27] were able to identify distinctive EEG networks of brain electrical activity, using EEG alpha coherence associated with each of the four personality orientations.

The theoretical model for each of the four GPO thinking styles is based on extensive qualitative research (depth interviews and focus groups), as well as quantitative surveys to establish their validity and reliability [9,10]. The four personality orientations are an alternative model to the dual processing systems [9,10,28], because the four GPO model more clearly identifies the nature of the logical slow deliberate rational processing (system 2) and adds more in-depth and specific explanation about the system 1, hypothesized to be fast, heuristic, or intuitive cognitive processing [28].

According to Gountas et al. [9,10,11], the four GPOs identify four distinctive traits based on fundamentally different modes of information processing. The broad traits of each of the four GPOs are as follows:

- *Thinking/Logical PO* (L): They primarily focus on semantic ideational thinking and rational conceptual thinking style; they tend to focus on generating new ideas, making sense of the why and how things work, and emphasizing the need to know rational explanations of phenomena. Logical ideational individuals focus on making sense of life based on the coherence of existing valid knowledge, and scientific findings. They are able to evaluate information objectively, based on factual criteria and logically analyzing, thinking through (often without emotional influences) the merits of concepts and events based on rational assessment [29,30].
- *Material/Pragmatic PO* (P): They focus on physical empirical evidence and knowledge structures. They are characterized by a strong emphasis on physical attributes, characteristics, and phenomena, which are measurable and tangible. They prefer to deal with the physically objective realities, amenable to objective investigation. They aim for material success, material possessions, and enjoying the physical advantages of the world. Materialists get immense pleasure from physical pleasures and the tangible attributes of products (often without concerns for social or emotional impacts). They rely heavily on the input of their somatosensory faculties to make decisions. Embodied type of learning and sense making is the dominant cognitive processing style.
- *Emotion/Feeling-Action PO* (E): They engage in experiential and action-based learning, understanding, and thinking processes. They tend to be action oriented, observing and planning actions, and have high levels of energy. High sensorimotor arousal, influences goal-motivated actions to satisfy their needs. They are usually social intelligent, competitively oriented toward social goal achievements. They are driven by a high sense of self-efficacy, and a ‘can do’ self-reliant attitude. They are able to makes sense and find solutions to various challenges through action-based strategies. Social learning, role playing, and trial error experiential learning generate ideas and thinking to make sense of their world. Experiential information underpins conceptual thinking and understanding of what decisions to make.
- *Intuitive/Imaginative PO* (I): They are creative types of thinkers, able to visualize information and able to think abstract constructs. Imaginative visualizers are characterized by a heightened level of sensitivity and awareness of unconscious perceptions regarding world phenomena. They tend to rely on visual, graphic, or animated types of learning stimuli. They are more likely to use imagination habitually to process information and problem solving. Abstract thinkers can develop complete solutions rather than following a sequential inductive step by step analytical approach. Information is mentally and visually processed with ease and counterfactual concepts emerge to solve problems. They can produce imaginative, novel creative ideas about world experiences and phenomena that are not empirically obviously detectable.

### Research Hypotheses

Previous research suggests that each orientation has a distinct frontal cortex organization with *Logical* thinking associated with left hemisphere and the *Intuitive/Imaginative* thinking style demonstrating a right hemisphere cortical organization and cognitive activation patterns [31]. Several fMRI studies suggest that ventromedial prefrontal cortex (vmPFC), orbitofrontal cortex (OFC), and posterior medial frontal cortex (pMFC) are associated with self-reflection types of responses and decision-making processes [20,32,33].

The current study was designed to expand our previous EEG research findings of the neural basis of the Gountas PO model, by using the blocked fMRI design [27]. The general hypothesis of this fMRI study was that the ventromedial prefrontal cortex, orbitofrontal cortex, and posterior medial cortex would be activated differentially by the four GPOs during a unique self–reflection paradigm, and in addition, would support the bilateral hemisphere and frontal and temporal-parietal relationships identified in the previous EEG study [27]. Based on previous literature, we also hypothesized that regions associated with various cognitive and emotional processes would be specifically related to each of the orientations. For example, the *Emotional PO* would be associated with regional emotional processing [34,35,36,37,38,39]. The *Logical PO* (the ideational logical reasoning) are hypothesized to produce stronger activation in prefrontal regions associated with executive function, [40,41]. The *Imaginative PO* (the creative visualizing thinkers), will demonstrate higher activations in the medial prefrontal cortex, inferior parietal cortex (including the temporoparietal junction), together with visual sensory brain regions [42,43,44,45]. Finally, the *Material/Pragmatic* type (the material/pragmatic rational thinkers) will produce stronger activations in the inferior parietal lobe (IPL) and association brain regions [19,41,42,46].

## 2. Materials and Methods

### 2.1. Participants

Informed and written consent was obtained from all participants (university students and community sample), according to the research protocols as approved by the University Human Ethics Committee. (SUHREC Project 2009/096) Initially, forty-eight (n = 48) participants were pre-tested with the Gountas Personality Orientation (GPO) instrument to determine their personality orientations and ensure equal numbers of at least ten participants of each of the four GPOs. A final sample of forty participants (n = 40, 10 for each PO) were selected for the fMRI study; with a mean age of 27.5 years and SD 7.6 years. The sample consisted of 22 females (20 right handed), and 18 males (16 right handed). The forty participants comprised four equal groups representing each of the four dominant personality orientations. All participants were self-screened for neurological conditions, ensuring that there was no history of head trauma, or any psychiatric disorders, or substance abuse, and appropriate current medication status to participate in the fMRI study.

### 2.2. Experimental Materials

The GPO Survey Assessment is a 39 question inventory identifying the four primary and secondary orientations of thinking styles. The GPO survey quantifies four personality orientation or broad traits that are associated with how people think (thinking style preferences), what types of information they naturally prefer to focus on (cognitive processing systems used), and what broad types of cognitive processes they go through [26,47]. The four GPOs are: *Emotion/Feeling-Action (E)*, *Material/Pragmatic (P) Intuitive/Imaginative (I)*, and *Thinking/Logical (L)*. Each participant completed the 39-question survey to group together the four phylogenetic groups of cognitive processing personality orientations.

For all fMRI experimental test recordings and analysis, participants were grouped based on their a priori dominant and secondary personality orientations as scored by the GPO instrument results. Based on the original paper by Gountas et al. [26], the survey results of the factor analysis for the four personality orientations produced a very robust Cronbach’s alpha for each orientation: *Thinking/Logical* α = 0.85, *Material/Pragmatic* α = 0.80, *Emotion/Feeling-Action* α = 0.83, and *Imaginative/Intuitive*, α = 0.85. Each of the orientations was also found to be independent from each other. To test our hypotheses, the fMRI results for each hypothesized PO (dominant and secondary orientation) were analyzed as four separate groups and therefore tested separately for differences from each of the other POs (e.g., *Emotion/Feeling-Action PO* vs. *Non-E/F PO*). This was done to improve and verify each PO group’s statistical significance for the fMRI results and each PO group was compared with the other three groups without the same PO traits. The final numbers in each grouping (with primary and secondary orientation) were: *Thinking/Logical* PO: (n = 19, *non-T* n = 21); *Intuitive/Imaginative* PO: (n = 19, *Non-I* n = 21); *Material/Pragmatic* PO: (n = 22, *Non-M* n = 18); *Emotion/Feeling-Action* PO: (n = 17, *Non-E* n = 23).

### 2.3. Neuroimaging fMRI Methodology

Participants were scanned using a Siemens 3T Tim Trio equipped with a 12-channel head coil at the Brain Research Institute (Melbourne, Australia). Each scanning session involved acquisition of a high-resolution T1-weighted magnetization-prepared gradient echo (MPRAGE) scan (192 sagittal slices; 0.9 mm isometric voxels; TR = 1900 ms, TE = 2.6 ms), that was used to register functional image activation into the standard stereotactic space defined by the Montreal Neurological Institute (MNI space). During each functional run, 139 gradient-echo, echo-planar T2*-weighted images (EPIs: TR = 3000 ms, TE = 30 ms, FoV = 216 × 216 mm, 72 × 72 imaging matrix, 44 interleaved axial slices, 3 mm isometric voxels) were acquired. The cognitive activation task was triggered by the onset of the sixth volume (volumes 1–5 were discarded), hence each experimental run was associated with 134 EPI images.

### 2.4. Neuroimaging Tasks

**Figure 1:**
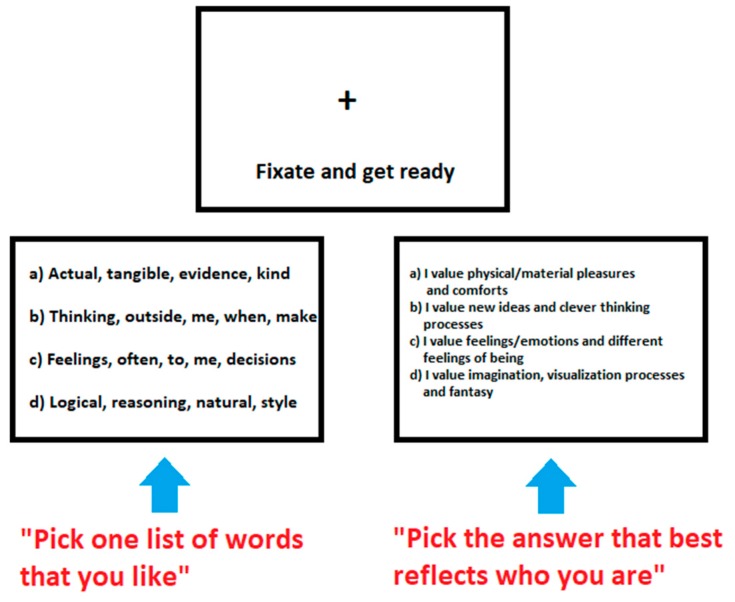
Three components of the block design protocol. The tasks were sectioned into three components of varying demand: *Fixation*-Rest (**top**), *Words* task (**left**) and *Sentences* self-reflection task (**right**). Examples of the words and sentences used are illustrated.

Stimulus delivery and recording of behavioral responses were controlled with E-Prime software (Psychology Software Tools Inc., Sharpsburg, PA, USA). The cognitive activation paradigm was presented in a typical block design comprising three tasks: a *Fixation task*, a *Word task*, and a *Sentence task* (or self-reflection task). The Word and Sentence tasks were constructed from words and questions based directly on the GPO psychometric instrument (see Figure 1). Participants had to make a ranking order choice between four alternative options corresponding to each of the four POs. Block onsets were locked to scan separate onsets, and each block lasted 21 s (i.e., seven scans or volumes). Each block was repeated 12 times (one trial), with a rest period mid-way through the protocol to secure against potential participant fatigue. Trials were repeated twice.

The Word and Sentence tasks were visually and physically similar (luminosity and spatial frequency) and based on the GPO survey to minimize any visual novelty effects. The participants were required to press (make a choice) one of four buttons for both tasks; each button corresponded to a separate choice of screen text (labelled as a, b, c, d, respectively). The requirement for the *Word task* was to read four lines of text and select the line of words they preferred most-least in a hierarchic sequence: a, b, c, or d. The requirement for the *Sentence task* was to read four lines of short sentences and choose which sentence best represented their own thinking style or cognitive processing (self-reflection). These trials and tasks were repeated as part of the scanning sequence (block design).

### 2.5. Neuroimaging and Statistical Analysis 

All aspects of image pre-processing and statistical analysis were performed using SPM8 (Wellcome Trust Centre for Neuroimaging) and associated toolboxes.

*a. Pre-processing*

Initially, slice-artifacts present in Echo-planar imaging (EPI) were repaired using *Artrepair* tools [48]. Functional images were then realigned to the first image acquired, and the T1 scan was co-registered to a mean realigned EPI computed during realignment. T1 images were subsequently normalized to the MNI template supplied with SPM8, and the parameters of this transformation were applied to realigned EPIs. Normalized EPIs were then smoothed using an 8 mm FWHM Gaussian filter.

*b. Modeling of neuroimaging data*

Participant level modeling of pre-processed fMRI data was performed on the image data acquired in the first run as a ‘localizer task’ and the remaining two runs (‘task’) separately, using the general linear model (GLM) approach in SPM8. Initially, separate box-car functions defining the onsets and durations of the three conditions (*fixation, word,* and *sentence* tasks) were convolved with the canonical hemodynamic response function (HRF) supplied in SPM8 for each analysis. Motion realignment parameters estimated during pre-processing were modeled as covariates of no interest, and after parameter estimation, contrasts were computed. A single subject multi-trial was done to estimate statistical power required for whether we needed 1, 2 or 3 trials. Based on these analyses, it was deemed that two trials were sufficient (7 × 12 = 84 scans). The SPM Statistical Design for Rest (*Fixate*), *Words*, and *Sentences* tasks was implemented accordingly (note threshold T = 3.11).

*c. Group level modeling*

Our analytic approach involved using the first run as a ‘localizer task’ from which regions of interest (ROIs) were determined via one-sample *t*-tests. Significant clusters of activation (thresholding was *p* < 0.001 uncorrected at the voxel level and *p* < 0.05 family-wise error (FWE) corrected at the cluster level) observed in these tests were set as ROIs. This was performed instead of doing non-independent analyses with voxel by psychometric correlations as suggested by Vul et al. [49]. Cluster corrections with family-wise error (FWE) and false discovery rate (FDR) were applied to cluster level and peak level data.

All consequent ROI processing (ROI building, signal extraction) was performed using *MarsBar* region of interest toolbox (Version 0.43) for SPM8 [50]. The mean signal intensity from each ‘scan’ and each ‘task’ run was extracted for each ROI. For each ROI, the signal time-course from the scan preceding each block (x_0_) until three scans following each block (i.e., 10 TRs) were averaged then baselined by subtracting the BOLD signal intensity from the first scan in each block (x_1_) from each TR (i.e., x_0_ − x_1_, x_1_ − x_1_, x_2_ − x_1_, …, x_10_ − x_1_) [51]. These time-courses were then averaged across groups and plotted as a function of TR. Brain activity associated with preponderant GPOs were examined in separate but complimentary analyses: (1) signal time-courses for each ROI were extracted from the remaining two runs and plotted for each group by condition, and (2) two-sample *t*-tests were computed to assess brain activation linked to GPOs.

## 3. Results

### 3.1. Personality Orientations and fMRI Activity

**Figure 2:**
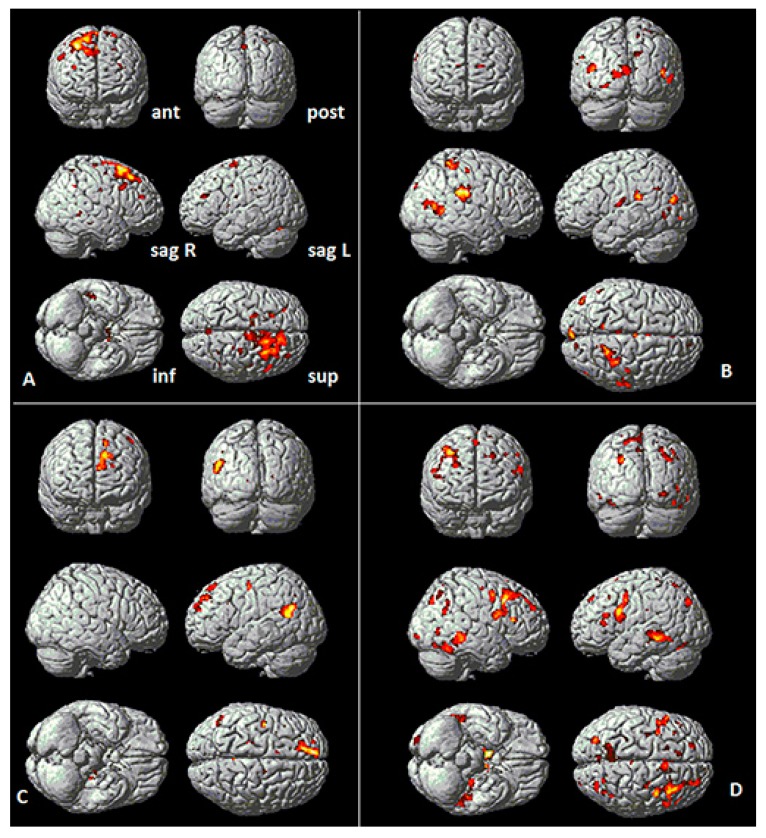
fMRI two-sample *t*-test Sentences versus Word Overall activation (threshold *p* < 0.05). Talairach coordinates for each significant regional difference are summarized in Table 1a,b. Note anatomical views are anterior (ant), posterior (pos), left sagittal (sag L), right sagittal (sag R), inferior (inf), and superior (sup). (**A**) (Top Left), **Emotion Type** (17 ***E*** *group* vs. 23 *Non-**E** group*). (**B**) (Top Right) **Imaginative Type** (n = 19 ***I*** *group* vs. 21 *Non-**I** group*). (**C**) (Bottom Left) **Logical Type** (19 ***L** group* vs. 21 *Non-**L** group*). (**D**) (Bottom Right) **Material Type** (22 *M group* vs. 18 *Non-M group*).

Self-reflection during the GPO experimental testing was localized using the block design for contrasting *Words* and *Sentences*. A group fMRI analysis was performed to examine each personality orientation: *Emotion/Feeling-Action (E)*, *Material/Pragmatic (P)*, *Intuitive/Imaginative (I)*, and *Thinking/Logical (L).* Using a two-sample t-test for contrasting groups, SPM analysis produced the fMRI images illustrated in Figure 2A–D. These demonstrate significant activation (*p* < 0.05) in different brain areas for each of the four orientations. Those with the specific PO orientation as either their dominant or secondary thinking style (Trait group) were contrasted against those without the PO, (Non-trait group), (see Table 1a,b). The effects of age, handedness, and gender were not found to be significant.

Additionally, the fMRI two sample t-test, for the Words vs. Sentences contrasts, demonstrate several other relationships. For example, the *Emotion/Feeling-Action* types have right frontal activity while the *Logical/Thinking* types have statistically more left frontal brain activity (see Figure 2A vs. Figure 2C). Interestingly, the stronger brain activation in the left hemisphere for the *Logical/Thinking* types contrasted with the *Emotion/Feeling-Action* and *Imaginative/Intuitive* types with more significant activation of the right hemisphere. The group analysis data (Trait PO vs. non-Trait PO) summarized in Table 1a,b, further highlight these different relationships.

### 3.2. Emotion/Feeling-Action Personality Orientation

The following Brodmann Areas 6, 8, 20, 30, 44, were significantly active (threshold *p* < 0.05, Family-Wise Error-FWE corrected) during the self-reflection phase of the reading tasks (Sentences vs. Words) for the participants with *E* PO (see Figure 2A and Table 1a). Figure 2A illustrates right frontal activation consistent with the hypothesis that *E* types use the right pFMC, which is involved in processing emotions [34]. Interestingly other regions associated with memory (hippocampus), planning complex movements, and representation of complex object features, and face perception were also activated, consistent with previous findings in emotion perception [53,54,55]. Compared to the other POs, this collection of brain regions for introspection is very specific to the *Emotion/Feeling-Action* PO. However, one area (BA30) was also active for the *Imaginative/Intuitive* PO but was restricted to the right hemisphere.

### 3.3. Intuitive/Imaginative Personality Orientation

For the *Intuitive/Imaginative* type, the following Brodmann Areas 2, 5, 11, 30, 37, 39, 41, 48 were significantly active (threshold *p* < 0.05), (see Figure 2B and Table 1a). This PO demonstrates an imaginative approach to decision-making, utilizing strategic thinking, intuition, emotions, and memory. This can be clearly seen by the fact that the orbitofrontal areas (BA39) and (BA11) were active (consistent with Yasuno et al. [19], supporting the hypothesis for the *Imaginative/Intuitive PO*. The various association regions involved in sensory and somatosensory were also involved (BA2, BA5 and BA41) together with face identification (BA37) and memory (BA48).

### 3.4. Logical/Thinking Personality Orientation

The key regions activated for this PO were those associated with logical, planning processes: the dorsolateral prefrontal cortex (BA9) and a region associated with integration of sensory and mnemonic information, the regulation of intellectual function, action, and working memory (BA46), (see Figure 2C and Table 1b). Other regions associated with decision-making, risk evaluation, social context, beliefs, and visual memory were also activated (BA18, 23, 25, 36, 37, and BA48). This is consistent with the hypothesis that the Logical trait would exhibit activation in the vmPFC and Dorsolateral Prefrontal Cortex, consistent with Saito et al. [40].

### 3.5. Material/Pragmatic Personality Orientation

This orientation primarily demonstrated regions of activation associated with empirical (mathematical) thinking and physical reward preferences, learning through factual information and analysis; and using empathy and emotion-based decision-making (anterior cingulate gyrus—BA32), (see Figure 2D and Table 1b). However, other regions associated with risk, fear, executive decision making (BA25), and identifying social context (BA36) were also significant for this type. This is consistent with the hypothesis that *Material/Pragmatic* traits are associated with vmPFC (BA25) activation [19,40]. Other regions activated were associated with complex physical object visual sensory processing (BA20) and sensory memory (BA48). This is consistent with the descriptors for the *Material/Pragmatic PO*, and that sensory information associated with decision-making and self-reward are important factors for this orientation.

The fMRI data analysis (Figure 2 and Table 1a, b) suggests that all hypotheses were supported; however, several additional brain regions were also activated for each PO. We observed that the *Emotion/Feeling* and *Intuitive/Imaginative POs* had their activations distributed to the right hemisphere, while *Thinking/Logical* types tended to have more left hemisphere distribution of activity. The *Material/Pragmatic* tended to have processes associated with both the left and right hemisphere.

### 3.6. Personality Orientations and ROI Activity-Localizer Scan

**Figure 3:**
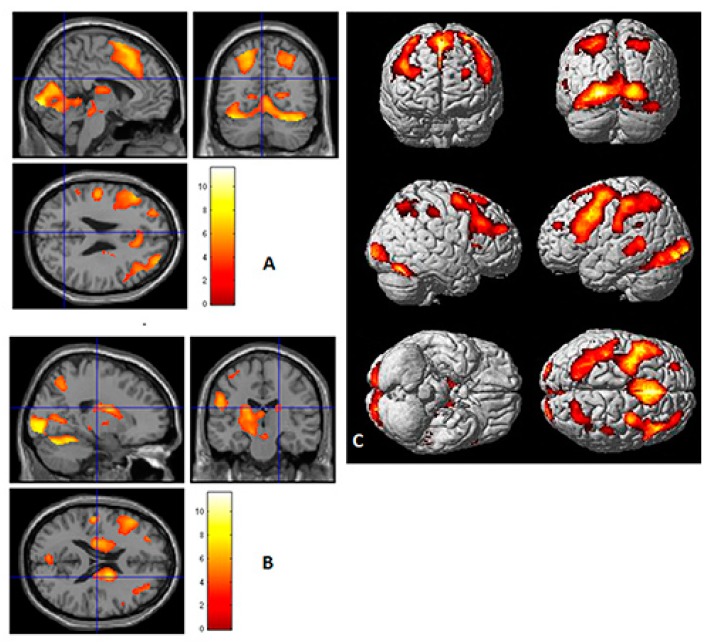
Localizer scan, region of interest analysis (ROIs). One sample *t*-test for “Sentences versus Baseline” overall activation. Note for n = 40, *t* = 3.312788 (*p* < 0.001). Twenty-two ROIs were identified by this approach (see Table 2). (**A**) One sample *t*-test Sentences vs. Baseline overall activation sectioned at the precuneus (sagittal, coronal, and transverse). (**B**) One sample *t*-test Sentences vs. Baseline overall activation sectioned (sagittal, coronal, transverse) at the level of the thalamus. (**C**) One sample *t*-test Sentences versus Baseline overall activation.

To understand the time-course characteristics of the task-related activation associated with each PO, *regions of interest* (ROIs) were identified using another approach, namely, MR time-course during localizer scans [51]. By analyzing all participant data together (n = 40) for *Sentences* versus *Words*, cortical and subcortical activations were identified as regions of interest (ROIs). This is illustrated in Figure 3, where a one-sample t-test was performed (note that T = 3.312788 for *p <* 0.001). Additional regions including subcortical were identified. Twenty-two ROIs were examined for further analysis. These are listed in Table 2.

**Figure 4:**
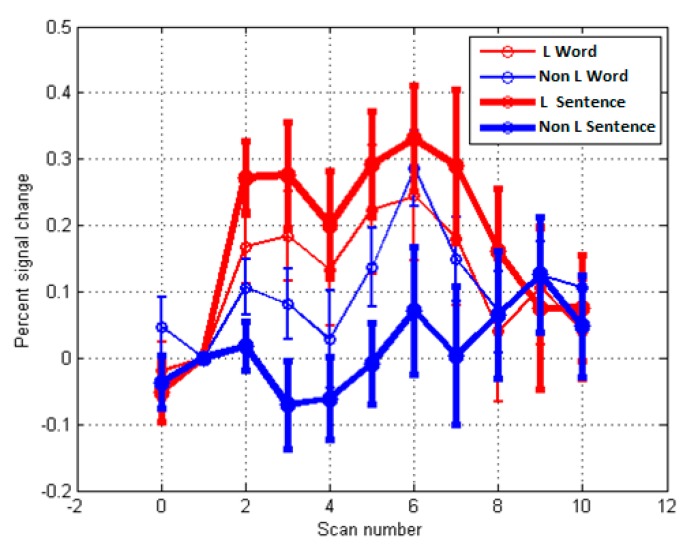
MR time-course during localizer activity averaged across participants for Sentences versus Word overall activation. (*p* < 0.05) for **Thinking/Logical PO**. The ROI highlighted is the left mid frontal cortex or Brodmann area 10; comparing the Logical group (red; n = 19) with the Non-Logical group (blue; n = 21). Note for Scans 3, 4, 5, (*p =* 0.011, 0.046, 0.044, respectively), *F* (1.38) = 7.137.

**Figure 5:**
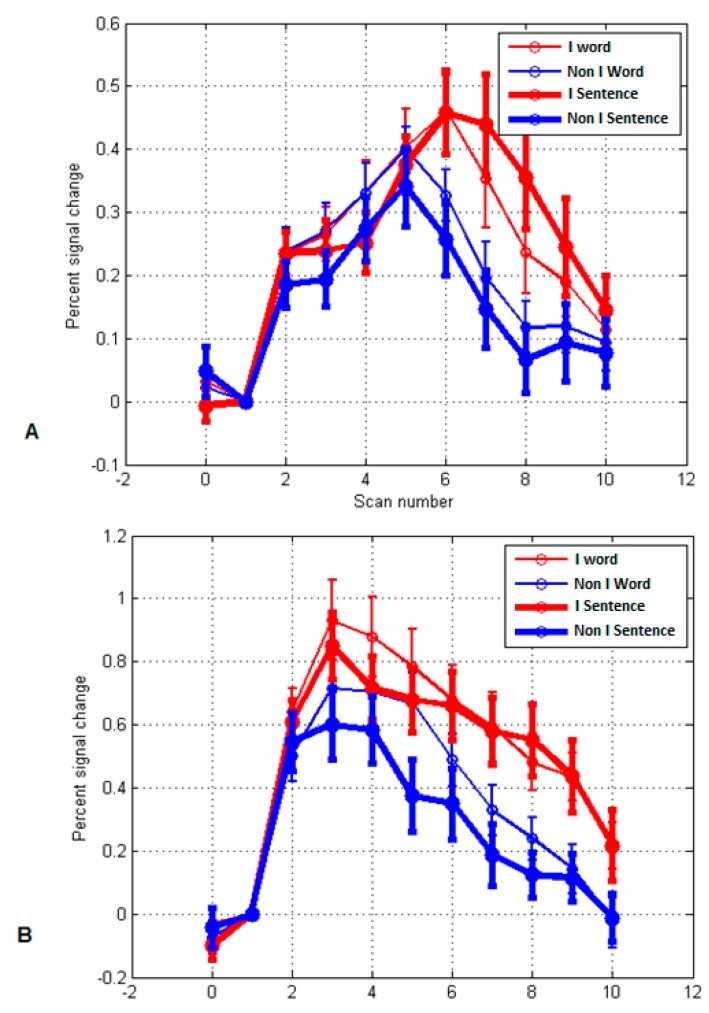
MR time-course during localizer activity averaged across participants for Sentences versus Word overall activation. (*p* < 0.05) for Intuitive/ Imaginative PO—ROI analysis, comparing Intuitive (red; n = 19) with Non-Intuitive (blue; n = 21). (**A**) Brodmann area 9 right dorsolateral prefrontal cortex (RDLPFC); for Scans 7, 8, 9, (*p* = 0.007, 0.001, 0.007), *F* (1.38) = 13.497, respectively. (**B**) Left cuneus or Brodmann area 17; for scan 6, 7, 8 (*p* = 0.032, 0.018, 0.013), *F* (1.38) = 6.760, respectively.

**Figure 6:**
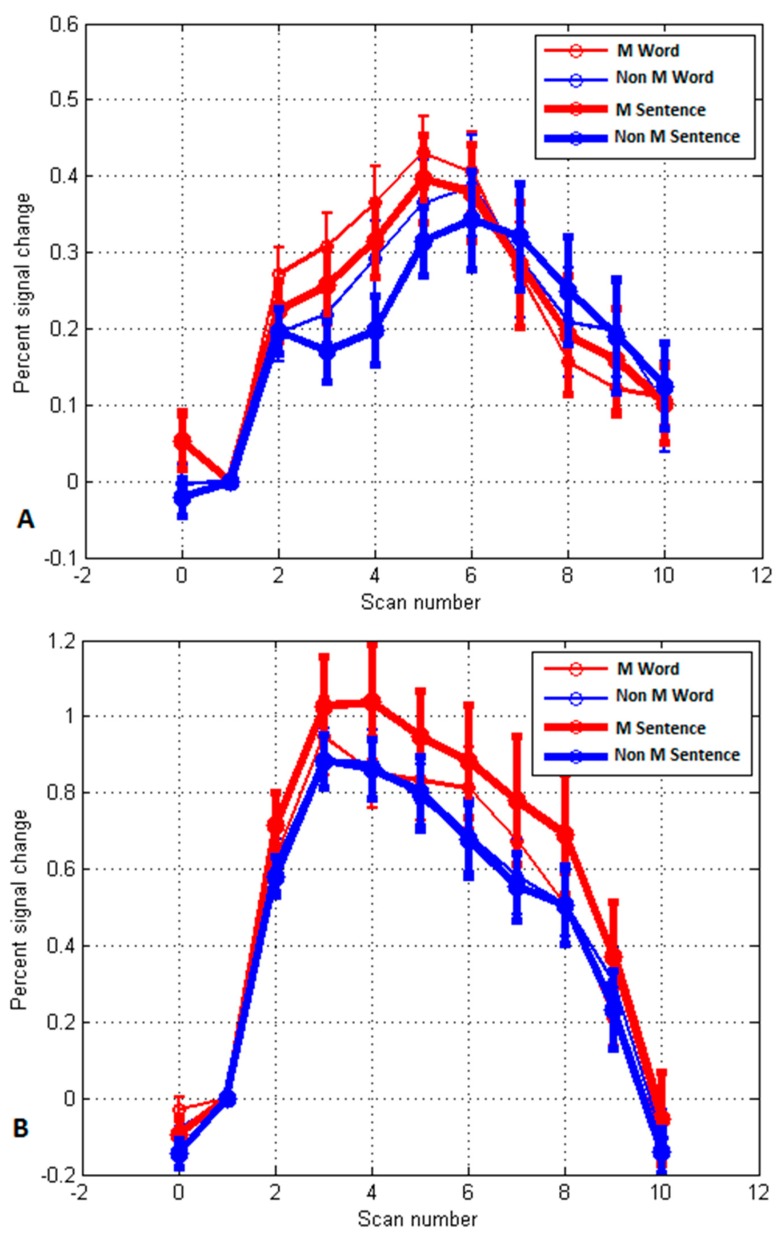
MR time-course during localizer activity averaged across participants for Sentences versus Word overall activation. (*p* < 0.05) for Material/Pragmatic PO—ROI time course analysis (22 *M-group* vs. 18 *non-M group*). (**A**) BA9 right DLPFC (Scan 3, where *p* = 0.038, *F* (1.38) = 4.290). (**B**) Superior parietal lobule, somatosensory association cortex (BA7), (Scan 6, where *p* = 0.033, *F* (1.38) = 4.890).

**Figure 7:**
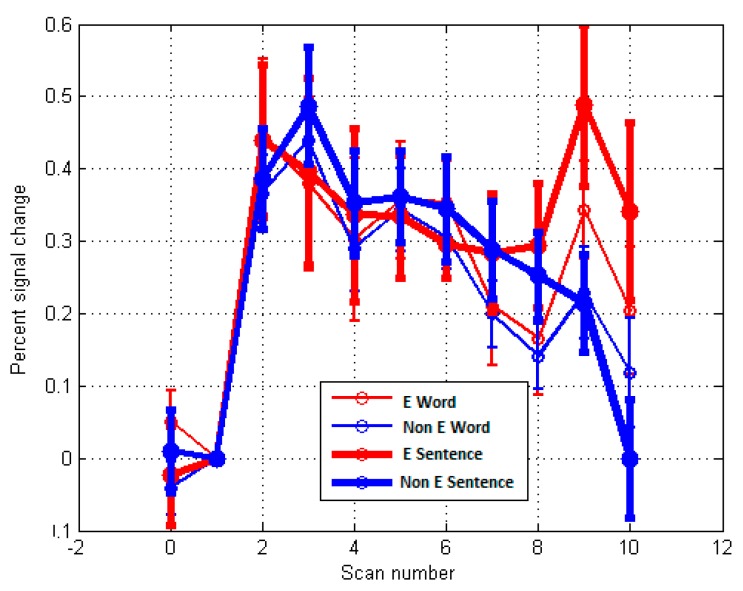
MR time-course during localizer activity averaged across participants for Sentences versus Word overall activation. (*p* < 0.05) for Emotion/Feeling-Action PO—ROI analysis (n = 17 E group versus n = 23 Non E group) for Brodmann area 9 (right hemisphere) where *p* = 0.032, *F* (1.38) = 4.955 for Scans 9 and 10.

The more significant frontal ROIs (from Table 2) for each PO were selected and produced as MR time-course images (see Figure 4, Figure 5, Figure 6 and Figure 7). Additionally, significant examples were also included for *Imaginative* and *Material* POs. Significant group effects were found with ROI (higher percentage signal change) versus PO (Table 3). Figure 4 illustrates the key ROI time-course associated with the *Thinking/Logical* types; the left mid-frontal cortex (BA10), which were significantly different (*p* = 0.011, 0.046, 0.044) for the average time course for Scans 3, 4 and 5, respectively, distinguished this *Logical* group from a *non-Logical* group with a higher signal change of this region.

Figure 5 again illustrates the fMRI two sample t-test Sentences versus Word overall activation and the average time course activation for the *Intuitive/Imaginative* PO. Sections A and B illustrate the time course (scans) for two significant regions; Brodmann area 9 right dorsolateral prefrontal cortex (RDLPFC); for Scans 7, 8, 9, (*p* = 0.007, 0.001, 0.007), *F* (1.38) = 13.497, respectively, and left cuneus or Brodmann areas 17; for Scans 6, 7, 8 (*p* = 0.032, 0.018, 0.013) *F* (1.38) = 6.760, respectively. During self-reflection regions associated with visualization, association, somatosensory, and emotional processes; left inferior frontal gyrus, left inferior parietal lobule, cuneus, and insula were significantly active (see Table 3). This is consistent with the PO description for this trait’s characteristics.

Similarly, in Figure 6, data for the *Material/Pragmatic PO* are illustrated for ROI BA7 (right hemisphere). Note that higher activity in Brodmann area 7 on right hemisphere where *p* = 0.033, *F* (1.38) = 4.890. This is consistent with the reliance on physical senses and somatosensory systems and a reliance on limbic systems (see Table 3, LMBG *p* = 0.004) for this PO. Figure 7 illustrates the fMRI activation and ROI time course for the right BA 9, where *p* = 0.032, *F* (1.38) = 4.955. Note that this is only significant for Scans 9 and 10, suggesting a difficulty in achieving activation during self-reflection.

## 4. Discussion

The aim of the current study was to explore the potential neural basis of personality related thinking styles using a self-reflection activation protocol. We hypothesized that this would elicit distinct significant activation in frontal regions such as the ventromedial prefrontal cortex, orbitofrontal cortex, and posterior medial cortex for each of the four traits. Primarily, this hypothesis was supported by a significant correlation between BOLD activation (ROI) and PO type; the *Emotional* type and the pMFC (BA6), the *Logical types* with vmPFC and dorsolateral prefrontal cortex (BA9), the *Imaginative* with OFC (BA11), and finally *Material/Pragmatic* with vmPFC (BA25).

Second, we also observed support for the bilateral hemisphere relationships identified in our previous EEG study [27,56]. Our current results suggest that different personality orientations with the corresponding thinking styles, during the fMRI experimental test introspection, are reflected in different neural organizations or preferred brain regional relationships. Each of the four PO groups demonstrated clear hemispherical lateralization differences and/or distinct regional recruitment during introspective decision-making. The *Imaginative* had significant activation of the visual sensory brain regions as expected, and the *Material/Pragmatic* with association in the somatosensory brain regions (Figure 5B and Figure 6B), consistent with GPO characteristics.

This work adds to the previous personality literature [15,17,32,33,57,58] and task design [57] by highlighting the specific roles and recruitment of specific brain regions associated with the characteristics/traits associated with each personality orientation during the self-reflection paradigm. Even though the study was constructed to investigate the specific role of the ventromedial prefrontal cortex, orbitofrontal cortex, and posterior medial cortex [20,32,33], regions associated with executive function—planning, strategy, emotions, memory, language and sensation association—were activated during self-reflection for each PO (Table 1a,b, Figure 2). We tentatively speculate that many of these additional regions may be a preferred or efficient process associated with each PO as part of their environmental and educational life experiences [26,29]. From these data, each orientation may have its own activation for processing self-awareness and decision making. Taken together, the specificity of the ROI for each *Personality Orientation* reflects specific introspection trait activation and perhaps the default mode networks as highlighted by Buckner et al. [16], with each PO demonstrating the most efficient network associated with their PO.

The *Emotional/Feeling-Action* PO, recruits regions associated with action observation, inferential thinking and planning, social learning, and emotion memory (i.e., BA 6, 8, 20, 30, 44). The cingulate gyrus (BA30) has been known to be involved in emotion formation and processing [52] and intense emotional reflection [59]. Visual association may also be a preferred mode of cognitive processing and it may be an important outcome of the interpretation of motor information for the *Emotion/Feeling-Action* types, allowing them to visualize action–emotion relationships during self-reflection (BA8, BA20).

The *Imaginative/Intuitive* PO demonstrated activation of regions known to be involved with creative thinking and counterfactual inventive thoughts (BA11, BA39). These regions are also involved in executive decision-making, understanding metaphors and abstract concepts [42,52]. This PO also had more regions activated in the right hemisphere (BA 2, 5, 11, 30, 37, 41, and 48), regions shown to be associated with higher executive decision making, memory, sensory associative processes [52], and imagined sensations [60]. During the self-reflection activation process, the *Imaginative/Intuitive* PO also activated regions associated with the fusiform and cingulate gyrus, consistent with previous imagination studies [42,60,61]. The *Material/Pragmatic* PO produced stronger activations with vMFC (BA25) brain regions, which have been reported to also process risk and semantic meaning, and activation in (BA32) related to physical reward-based learning (see Table 1b). This is consistent with the behavioral characteristics associated with the Material/Pragmatic PO type. Similarly, the *Logical/Thinking PO* activated brain regions associated with executive decision-making, consolidation of ideas and organizing information (BA 46, 9, 10, and 23), (see Table 1b).

To further identify these distinct PO regional relationships, localizer scans associated with ROIs (see Figure 3 and Table 2) calculated from the whole cohort were applied [36]. Moreover, by comparing the associated fMRI localizer scans, twenty-two regions of interest (ROI) were identified. The scanning time or average time-course of activation was calculated to illustrate the ROI activation for each dominant PO’s frontal and associated regions significant activations by scan (see Figure 4, Figure 5, Figure 6 and Figure 7). This ROI data analysis using Brodmann’s functional localization summary [52] adds further weight to the specific regions activated, which support the hypothesized behavioral characteristics of each PO.

According to these ROI analyses, the *Logical/Thinking* PO was represented by specific activity in the left mid frontal gyrus (see Figure 4) or Brodmann area 9 during the self-reflection task analyses (i.e., Sentences > Words-Introspection). The data suggest that those with *Logical PO* dominant personality orientation tend to rely on networks normally associated with strategic planning and higher executive functions, located in the left dorsolateral prefrontal cortex [62]. As listed in Table 3, this specifically occurs during the average time course activation during Scans 3 to 5, demonstrating the value of investigating time course information associated with cognitive processes. Adelstein et al. [17] also demonstrated similar activation for specific personality traits such as *Openness to Experience*, specifically the intellect facet of the NEO-PI [63]. Interestingly, the *Logical* PO may have similar neural processes to the intellect facet of the Openness to Experience.

The *Emotion/Feeling-Action* PO, however, is best represented with significant activation in the left and right dorsal lateral prefrontal cortex, frontal regions (BA6, BA47), and the left caudate nucleus (Table 3). Those who do not have this PO had significantly different regions activated in the frontal, parietal, occipital, and limbic regions, consistent with the findings associated with low emotional intelligence [64], suggesting a low efficiency in processing emotional information. The *Emotion/Feeling-Action* PO has more right activity than the left, consistent with previous research associated with emotional processing [65]. However, this was further supported by a recent study by Haas et al. [18] who reported increased BOLD activity in the right temporo-parietal cortex during performance of an emotion attribution task. Interestingly, these participants were also able to engage the emotion networks more quickly than others who scored low in the agreeableness trait. These two traits may be associated by activating similar brain region processes.

Similarly, this study revealed a number of regions activated for the *Material/Pragmatic* PO. This PO demonstrated brain activations associated with both the left and right hemispheres and amygdala. This may be consistent with the findings from previous fMRI studies associated with the NEO-PI’s *Extraversion* facet as reported by Omura et al. [66] whereby similar regions were activated as for the *Material* PO. This conjecture is supported by the reported similarities in personality characteristics associated with *Material* PO and Extraversion and Neuroticism [67]. Similar findings were also reported with respect to low social skills [3]. The *Imaginative/Intuitive* PO produced distinct ROI activities distributed at the right hemisphere and involving more networks in the frontal, parietal, occipital, and limbic areas than any other PO (see Table 3). The *Imaginative* PO related findings suggest that many resources are available for efficient processing of different types of information (e.g., logical, emotional and imagery/pictorial). This is consistent with studies in sensation and perception [61], memory and intelligence [68], and trait and identity coupled networks, as reported by Hassabis et al. [69]. Regions associated with visual processing (BA 16, 17, cuneus) tend to be more significantly active in the *Imaginatives*, which is consistent with the literature supporting the role of imagination and visual imagery [61]. However, unlike *Imaginative PO*, the *Logical PO* tends to have more left hemisphere distribution of activity and centered in the left prefrontal cortex; the dorsolateral prefrontal cortex. This region is known for higher executive functions such as integrating complex relations [70].

The fMRI data highlighted distinctive characteristics across all four orientations in the ventromedial prefrontal cortex (VMPFC) and orbitofrontal cortex (OFC). In a review by Wagner et al. [71], they suggested that the VMPFC is more active during periods of self-evaluation, while the OFC becomes more active during unrealistic or overconfident judgments of self-evaluation. The *Emotion/Feeling-Action* PO also demonstrated higher OFC activity. The M and *L* PO did demonstrate VMPFC activity consistent with self-perception and self-representation neural systems highlighted by Wagner et al. [71]. This study demonstrates the value of using a self r-reflection paradigm, which is able to “tease” out the preferred networks associated with each orientation. However, it has also produced findings that give further insight into self-image and the influence of personality orientations. Neuroimaging results were also consistent with Johnson et al. [32,33], where medial prefrontal and posterior medial cortex activation occurred in association with self-reflection, instrumental, or experiential self-reflection, respectively. Our data suggest that the narrative or idea of one’s self-image seems related or influenced by their personality orientation or thinking style. This has implications with respect to self-insight [72], social interaction, and meta-consciousness [73]. The paradigm could be useful in future investigation of the nature of self-image and inward and outward directed focus of one’s sense of self. According to Hixon and Swann [72], self-introspection is useful in improving insight into oneself, and may be useful for improving the outcomes of various psychological interventions. We have designed a paradigm that is able to identify and measure these processes and potential influences.

## 5. Conclusions

In the current study, we observed distinctive brain region activation associated with self-reflection, corroborating previous research [27]. However, the current study further revealed more spatial and functional detail by use of the statistical parametric mapping fMRI techniques, illustrating the specificity of the ROIs for each *Personality Orientation.* These findings clearly add to the current personality networks literature [20,71] and highlight the activation reflecting the cognitive substrates used to make introspective decisions.

Nonetheless, caution needs to be applied in interpreting the data because of the use of reverse inferences [74,75]. Given the exploratory nature of this study, the reported interpretation of the data should be considered as a hypothesis generated for both neurotypical and clinical cohorts; for personality disorders as well as for understanding how individuals can react to emotional stimuli [76] based on their personality orientations and traits.

## Figures and Tables

**Table 1:** (**a**) Group analysis (trait versus non-trait) contrast of *Emotion/Feeling-Action PO* and *Imaginative/Intuitive PO*; region by Talairach coordinates, Brodmann area (BA), hemisphere (H), and function (significant activations *p* < 0.05). Note definitions of common functions based on previous work [52]. (**b**) Group analysis (trait versus Non-trait) contrast of *Material/Pragmatic PO* and *Logical/Thinking PO*; region by Talairach coordinates, Brodmann area (BA), hemisphere (H), and function (significant activations *p* < 0.05). Note definitions of common functions, see [52].

(**a**)			
***Talairach Coordinates (x, y, z)***	***BA***	***H***	***Region—Functional Role (Brodmann Areas)***
***Emotion/Feeling-Action***			
16, 12, 68 14, 2, 54 −36, −4, 16 −24, −2, 68	6	R R L L	Prefrontal motor, supplementary motor area—planning complex movements—error analysis.
30, 12, 62	8	R	Includes frontal eye fields—control of visual attention, eye movements.
−30, −16, −18 −22, −8, 40	20	L L	Inferior temporal gyrus. Associated visual processing.
−14, −34, −20	30	L	Cingulate cortex—part of the limbic lobe. Executive functions of emotion formation and processing, learning and memory.
38, −8, 34	44	R	Hippocampus—memory formation & function.
***Intuitive/Imaginative***			
30, −45, 54	2	R	Postcentral Gyrus—Somatosensory processing, texture, size & shape.
16, −52, 56	5	R	Somatosensory Association.
32, 30, 22	11	R	Orbitofrontal cortex (OFC)—executive decision—making, understanding metaphors and invention.
18, −30, −18	30	R	Cingulate cortex—part of the limbic lobe. Executive functions of emotion formation and processing, learning and memory.
−40, −44, −16 −32, −48, −16 42, −64, 10	37	L L R	Fusiform Gyrus—face and body recognition, within—category identification.
−42, −78, 20	39	L	Orbitofrontal cortex (OFC)—executive decision—making, understanding metaphors and invention.
42, −38, 20	41	R	Primary Auditory Cortex—association sound.
44, −22, 24 50, −28, 28	48	R R	Retrosubicular area—Hippocampus-memory.
(**b**)			
***Talairach Coordinates x, y, z***	***BA***	***H***	***Region—Functional Role (Brodmann Areas)***
***Material/Pragmatic***			
42, 2, 22 34, 15, 42	20	R	Inferior temporal gyrus. Associated visual processing (complex objects).
50, 6, 22	21	R	Middle temporal gyrus. Processes include contemplation, facial recognition and word meaning.
12, −4, −4	25	R	Ventral medial prefrontal—risk & fear processing
−12, 26, 28	32	L	Anterior cingulate cortex (ACC). Involved in reward anticipation, decision—making, empathy and emotion and reward—based learning.
−20, 6, 38	36	L	Parahippocampal gyrus. Involved in identifying social context, encoding and recognition of scenarios.
−20, 10, 26	48	L	Retrosubicular area—Hippocampus—memory
***Logical/Thinking***			
−14, 38, 54	9	L	Dorsolateral prefrontal cortex. Involved in executive processes associated with motor planning, organization, and regulation, integration of sensory information and working memory.
12, −48, −4	18	R	Visual area V2. Involved in visual memory.
−4, −48, 30	23	L	Posterior cingulate cortex. Involved in the capacity to understand what other people believe, default mode & awareness.
−8, 16, 16	25	L	Ventral medial prefrontal—decision processing associated with risk & fear.
−26, −8, 12	34	L	Entorhinal cortex (EC). Involved in memory and navigation, memory formation, consolidation organization.
30, −2, 22	36	R	Parahippocampal gyrus. Involved in identifying social context, encoding and recognition of scenarios.
−28, −30, 0 −20, −46, 6	37	L L	Fusiform Gyrus. Involved in face and body recognition & identification.
−44, −64, 28	46	L	Dorsolateral prefrontal cortex. Involved in executive processes associated with motor planning, organization, and regulation, integration of sensory information and working memory.
28, 0, 14	48	R	Retrosubicular area—Hippocampus. Involved in memory formation.

**Table 2:** Summary of regions of interest (ROI): 22 ROIs were identified from data associated with Figure 3. Note (*p* < 0.05) (Talairach coordinates Brodmann area (BA), hemisphere (H)).

ROI	Talairach Coordinates (x, y, z)	H	Region (Nearest Gray Matter)	BA
1	−30, 50, 24	L	Left Cerebrum, Frontal Lobe, Superior Frontal Gyrus, Anterior Prefrontal Cortex	10
2	32, 52, 26	R	Right Cerebrum, Frontal Lobe, Superior Frontal Gyrus, Dorsolateral Prefrontal cortex	9
3	6, 14, 50	R	Right Cerebrum, Frontal Lobe, Superior Frontal Gyrus, Premotor Cortex	6
4	30, −64, 52	R	Right Cerebrum, Parietal Lobe, Superior Parietal Lobule, Somatosensory Association Cortex	7
5	−28, −56, 54	L	Left Cerebrum, Parietal Lobe, Superior Parietal Lobule, Somatosensory Association Cortex	7
6	−18, −96, −2	L	Left Cerebrum, Occipital Lobe, Cuneus, primary visual cortex	17
7	12, −92, −8	R	Right Cerebrum, Occipital Lobe, Inferior Occipital Gyrus	17
8	−48, 24, 24	L	Left Cerebrum, Frontal Lobe, Middle Frontal Gyrus, Dorsolateral Prefrontal cortex	46
9	46, 30, 30	R	Right Cerebrum, Frontal Lobe, Middle Frontal Gyrus, Dorsolateral Prefrontal cortex	9
10	32, −2, 62	R	Right Cerebrum, Frontal Lobe, Middle Frontal Gyrus, Premotor Cortex	6
11	−36, −2, 60	L	Left Cerebrum, Frontal Lobe, Middle Frontal Gyrus, Premotor Cortex	6
12	−14, −6, 16	L	Left Cerebrum, Sub-lobar, Caudate	
13	16, −4, 18	R	Right Cerebrum, Sub-lobar, Caudate	
14	10, −78, 8	L	Left Cerebrum, Occipital Lobe, Cuneus, primary visual cortex	17
15	−42, −36, 44	L	Left Cerebrum, Parietal Lobe, Inferior Parietal Lobule	40
16	−10, −22, −8	L	Left Brainstem, Midbrain, Thalamus, Gray Matter, Medial Geniculum Body	
17	−39, −72, −12	L	Left Cerebrum, Frontal Lobe, Inferior Frontal Gyrus, inferior prefrontal gyrus	47
18	37, −64, −20	R	Right Cerebellum, Posterior Lobe, Declive	
19	32, 25, 7	R	Right Cerebrum, Frontal Lobe, Inferior Frontal Gyrus, Broca’s area	45
20	−41, 23, 7	L	Left Cerebrum, Frontal Lobe, Inferior Frontal Gyrus, insular	13
21	−9, −15, −17	L	Parahippocampal gyrus, limbic	-
22	32, 21, 9	R	Sub-lobar, insula	13

**Table 3:** PO ROI Time course analysis: significant PO trait group effects—ROI (Higher activity—% Signal Change) Note: for between group statistics—* *p* < 0.05, ** *p* < 0.001).

*Brodmann Area/ROI*	*Volumes/Period*	*Probability* (*p* < 0.05)	*F* (1.38)
***Logical/Thinking***			
10—Left Mid frontal cortex (LmFC)	3	0.011 *	7.137
	4	0.046 *	4.245
	5	0.044 *	4.322
***Emotion/Feeling-Action***			
46—Left Dorsolateral Prefrontal Cortex	10	0.044 *	4.342
9—Right Dorsolateral Prefrontal Cortex	9	0.032 *	4.955
6—Middle Right Superior Frontal gyrus	10	0.049 *	4.151
Left Thalamus (caudate nucleus)	10	0.023 *	5.585
47—Left Inferior Frontal Gyrus	10	0.004 *	4.537
***Material/Pragmatic***			
Left Medial Geniculum	5	0.033 *	4.890
***Intuitive/Imaginative***			
9—Right Dorsolateral Prefrontal Cortex	6	0.032 *	4.928
	7	0.018 *	6.072
	8	0.013 *	6.760
6—Right Superior Frontal gyrus	6	0.011 *	7.176
	7	0.030 *	5.096
	8	0.024 *	5.495
	9	0.032 *	4.946
	10	0.028 *	5.223
7—left Superior Parietal Lobule somatosensory	7	0.004 **	9.502
	8	0.002 **	10.766
	9	0.033 *	4.875
	10	0.044*	4.320
17—Left Primary Visual Cortex	7	0.029 *	5.118
	8	0.015 *	6.425
	9	0.036 *	4.724
17—Cuneus	7	0.015 *	6.430
	8	0.006 **	8.482
	9	0.006 **	8.582
15—Left Inferior Parietal Lobule	7	0.018 *	6.103
	8	0.003 **	10.164
	9	0.007 **	8.025
17—Left Inferior Frontal Gyrus	7	0.007 **	8.107
	8	0.001 **	13.497
	9	0.007 **	8.241
Parahippocampal gyrus, Limbic	6	0.032 *	4.978
16—Left Primary Visual Cortex (Cuneus)	9	0.020 *	5.917
	10	0.032 *	4.958
Right Caudate	7	0.010 *	7.290
	8	0.018 *	6.059
13—Right Insula	6	0.048 *	4.159
